# Recombinant multiepitope proteins expressed in *Escherichia coli* cells and their potential for immunodiagnosis

**DOI:** 10.1186/s12934-024-02418-w

**Published:** 2024-05-22

**Authors:** Ana Alice Maia Gonçalves, Anna Julia Ribeiro, Carlos Ananias Aparecido Resende, Carolina Alves Petit Couto, Isadora Braga Gandra, Isabelle Caroline dos Santos Barcelos, Jonatas Oliveira da Silva, Juliana Martins Machado, Kamila Alves Silva, Líria Souza Silva, Michelli dos Santos, Lucas da Silva Lopes, Mariana Teixeira de Faria, Sabrina Paula Pereira, Sandra Rodrigues Xavier, Matheus Motta Aragão, Mayron Antonio Candida-Puma, Izadora Cristina Moreira de Oliveira, Amanda Araujo Souza, Lais Moreira Nogueira, Mariana Campos da Paz, Eduardo Antônio Ferraz Coelho, Rodolfo Cordeiro Giunchetti, Sonia Maria de Freitas, Miguel Angel Chávez-Fumagalli, Ronaldo Alves Pinto Nagem, Alexsandro Sobreira Galdino

**Affiliations:** 1 Microorganism Biotechnology Laboratory, National Institute of Science and Technology on Industrial Biotechnology (INCT-BI), Federal University of São João Del-Rei, Midwest Campus, Divinópolis, 35501-296 Brazil; 2 Department of Biochemistry and Immunology, Federal University of Minas Gerais, Belo Horizonte, 31270-901 Brazil; 3 Computational Biology and Chemistry Research Group, Vicerrectorado de Investigación, Universidad Católica de Santa María, Arequipa, 04000 Peru; 4 Biophysics Laboratory, Institute of Biological Sciences, Department of Cell Biology, University of Brasilia, Brasília, 70910-900 Brazil; 5 Bioactives and Nanobiotechnology Laboratory, Federal University of São João Del-Rei, Midwest Campus, Divinópolis, 35501-296 Brazil; 6 Postgraduate Program in Health Sciences, Infectious Diseases and Tropical Medicine, Faculty of Medicine, Federal University of Minas Gerais, Belo Horizonte, 30130-100 Brazil; 7 Laboratory of Biology of Cell Interactions, National Institute of Science and Technology on Tropical Diseases (INCT-DT), Department of Morphology, Federal University of Minas Gerais, Belo Horizonte, 31270-901 Brazil

**Keywords:** Recombinant multiepitope proteins, *Escherichia coli*, Bioinformatics, Biophysical analysis, Immunodiagnosis

## Abstract

Recombinant multiepitope proteins (RMPs) are a promising alternative for application in diagnostic tests and, given their wide application in the most diverse diseases, this review article aims to survey the use of these antigens for diagnosis, as well as discuss the main points surrounding these antigens. RMPs usually consisting of linear, immunodominant, and phylogenetically conserved epitopes, has been applied in the experimental diagnosis of various human and animal diseases, such as leishmaniasis, brucellosis, cysticercosis, Chagas disease, hepatitis, leptospirosis, leprosy, filariasis, schistosomiasis, dengue, and COVID-19. The synthetic genes for these epitopes are joined to code a single RMP, either with spacers or fused, with different biochemical properties. The epitopes’ high density within the RMPs contributes to a high degree of sensitivity and specificity. The RMPs can also sidestep the need for multiple peptide synthesis or multiple recombinant proteins, reducing costs and enhancing the standardization conditions for immunoassays. Methods such as bioinformatics and circular dichroism have been widely applied in the development of new RMPs, helping to guide their construction and better understand their structure. Several RMPs have been expressed, mainly using the *Escherichia coli* expression system, highlighting the importance of these cells in the biotechnological field. In fact, technological advances in this area, offering a wide range of different strains to be used, make these cells the most widely used expression platform. RMPs have been experimentally used to diagnose a broad range of illnesses in the laboratory, suggesting they could also be useful for accurate diagnoses commercially. On this point, the RMP method offers a tempting substitute for the production of promising antigens used to assemble commercial diagnostic kits.

## Introduction

Recombinant multiepitope proteins (RMP) are the result of epitopes joining to form a single molecule that does not exist in nature [1, 2]. A study by Dipti et al. (2006) is thought to be the first one to define an RMP as a molecule that contains linear, immunodominant, and conserved epitopes that are connected through linkers [1]. Since then, the commercial use of these molecules has gained market space with many applications related to human and animal health, such as the development of vaccines and diagnostic devices [3–7]. In fact, the global market of recombinant proteins is expected to grow by 12% from 2022 to 2030, with 2030 revenue estimated at USD 5.09 billion [8].

To form a new RMP, the first step involves selecting the epitopes to be used. This can be performed in several ways, such as choosing epitopes that have already been characterized as immunodominant in the literature [9–11], and through bioinformatics analyses [12–14]. Bioinformatics analysis identifies a pathogen’s antigens using computational analyses of its genome, without the need to manipulate the microorganism [15]. This implies a reduction in costs and research time, and minimizes the use of animals, proving to be an effective method for better targeting in in vitro and in vivo experiments [14, 16–19]. As much as it is an already consolidated area, and its importance demonstrated by several studies, the importance of bioinformatics became evident during the COVID-19 pandemic, during which it was necessary to select epitopes as quickly and inexpensively as possible to develop vaccines and diagnostic tests, increasing the number of studies using these analyses [20–24].

**Fig. 1:**
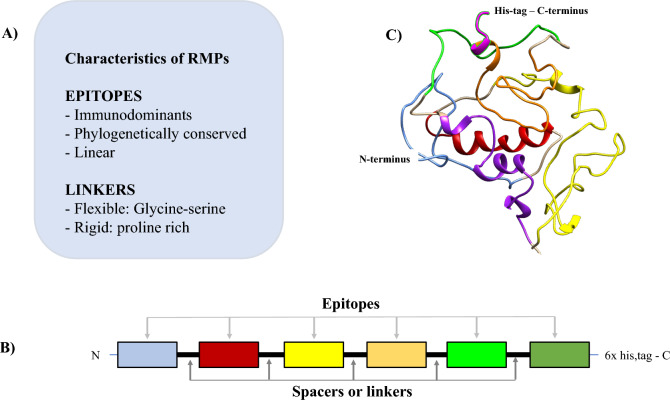
Design of a putative recombinant multiepitope protein. **A** General characteristics of majority RMPs available on literature. **B** According to the information presented in (**A**), a linear structure can be rationally drawn by the researchers. **C** The RMP 3D structure can be visualized from amino acid’s sequence by programs

Designing the new RMP includes many steps, such as: (*i*) selection of how many epitopes will be used; (*ii*) selection of spacing linkers between each epitope; (*iii*) tag selection to allow for better heterologous expression and purification; and (*iv*), evaluation of the physicochemical RMPs parameters [2] (Fig. 1). The next step is to choose the most appropriate host organism for RMP expression. The recombinant protein technology has become available worldwide, and several expression platforms are now available [25]. Among them, *Escherichia coli* became the most popular expression platform due to its relative simplicity, quick and inexpensive cultivation, and the availability of various compatible biotechnological tools [26]. However, this expression system comes with several drawbacks, such as the production of the protein in inclusion bodies and the absence of post-translational modifications [26–28]. To overcome these problems, expression platforms, such as yeast, insect, and mammalian cells, have been developed, and the choice of the best platform depends on the characteristics of each RMP and its applicability [25–27, 29]. Nevertheless, the recombinant protein technology is an efficient method for obtaining antigens at a relatively low cost that favors a more cost-effective production.

Among their diverse applications, RMPs have been widely used, experimentally and commercially, to diagnose a wide range of human and animal diseases through tests, such as enzyme-linked immunosorbent assay (ELISA), immunofluorescence assays, and lateral flow tests. For example, RMPs has been used in Chagas IgG-ELISA^®^ (NovaTec Immunodiagnostica GmbH; Dietzenbach, Germany) and Chagas Detect^™^ *Plus* (CDP) Rapid Test (InBios; Seattle, Washington, USA) commercial kits for Chagas disease detection. In this sense, these molecules offer advantages in the field of immunological diagnosis, such as increased sensitivity and specificity, which can improve diagnostic accuracy [1, 13]. Additionally, RMPs can be mass-produced, which facilitates diagnostic device standardization [13, 30–32]. The aim of this review was to select only studies using the nomenclature “recombinant multiepitope protein” and summarize all the research that used RMPs in diagnosing human and animal diseases, as well as explore significant issues surrounding this technology.

## RMPs applied in disease diagnosis

### RMPs applied in the diagnosis of diseases caused by bacteria

Currently, more than one thousand species of bacteria have been described as being pathogenic to vertebrate hosts [33, 34]. The most recent estimates have shown that more than seven million deaths were caused by bacterial infections in 2019, representing the second leading cause of global deaths [35]. Additionally, antibiotic resistance currently represents one of the greatest threats to global health [36, 37]. In this sense, the ability to provide a rapid and accurate diagnosis is essential for correct clinical conduct, improving the effectiveness of treatments and helping in antibiotic misuse [38]. RMPs has been applied in the experimental diagnoses of infections caused by bacteria and Table 1 summarizes the main points of these studies.

**Table 1:** RMPs applied in the diagnosis of diseases caused by bacteria

Disease/causative agent	RMP name	Host for protein expression	Serological assay	Samples (n)	Results	Reference/country
Human tuberculosis/*Mycobacterium tuberculosis*	TbF6	*E*. *coli* cells	ELISA	272 tuberculosis-positive serum samples/339 serum samples from healthy individuals	Sensitivity: 71.2–86% Specificity: 94.7–98.3%	[39]/USA
Human leptospirosis/*Leptospira interrogans*	r-LMP	*E. coli)* BL21(DE3) pLysS cells	ELISA	156 leptospirosis-positive serum samples/10 serum samples from healthy individuals/24 serum samples from non-leptospirosis diseases	r-LMP was recognized by all positive samples, without cross-reactions	[40]/China
Human leprosy/*Mycobacterium leprae*	PADL	*E*. *coli* HMS-174 cells	ELISA	54 leprosy- positive serum samples/18 serum samples from healthy individuals	PADL was recognized by positive samples	[41]/USA
Human tuberculosis/*Mycobacterium tuberculosis*	Fusion protein antigen	*E. coli* BL21(DE3) cells	ELISA	171 tuberculosis-positive serum samples/48 serum samples from healthy individuals/38 serum samples from non-tuberculosis diseases	Sensitivity: 42.1% Specificity: 89.5%	[42]/China
Human tuberculosis/*Mycobacterium tuberculosis*	PstS1-LEP	*E. coli* BL21(DE3) cells	Indirect ELISA	442 tuberculosis-positive serum samples/102 serum samples from healthy individuals/75 serum samples from non-tuberculosis diseases	Sensitivity: 42.1–82% Specificity: 72.2–95.2%	[43]/China
Human brucellosis/*Brucella* spp.	Recombinant protein	*E. coli* BL21(DE3) cells	Indirect ELISA	146 brucellosis-positive serum samples/20 serum samples from healthy individuals/82 serum samples from non-brucellosis diseases	Sensitivity: 88.89% Specificity: 85.54%	[44]/China
Human Lyme disease/*Borrelia burgdorferi*	A/C-2, A/C-4, and A/C-7.1	*E*. *coli* (XL1-Blue) cells	ELISA	169 Lyme-positive serum samples/5 serum samples from healthy individuals	A/C-2 Sensitivity: 80.17% Specificity: 52.83% A/C-4 results not shown A/C-7.1 Sensitivity: 91.37% Specificity: 73.58%	[45]/Slovakia
Human and goat brucellosis/*Brucella* spp.	rOmp	*E. coli* BL21(DE3) cells	Indirect ELISA	40 human and 31 goat brucellosis-positive serum samples/20 healthy human and 20 healthy goat serum samples	rOmp was recognized by human and goat positive samples	[46]/China
Goat brucellosis/*Brucella* spp.	rMEP	–	Indirect ELISA	57 brucellosis-positive serum samples/36 healthy goat serum samples	Sensitivity: 96.49% Specificity: 94.44%	[47]/China
Bovine and goat brucellosis/*Brucella* spp.	Multi-epitope fusion protein	*E*. *coli* BL21 cells	Indirect ELISA	116 and 140 goat brucellosis-positive serum samples/75 healthy bovine and 54 healthy goat serum samples	Bovine Sensitivity: 97.85% Specificity: 96.61% Goat Sensitivity: 98.85% Specificity: 98.51%	[48]/China
Human brucellosis/*Brucella* spp.	Fusion protein	*E. coli* BL21(DE3) cells	nano-p-ELISA	121 brucellosis-positive serum samples/50 serum samples from healthy individuals/40 serum samples from non-brucellosis diseases	Sensitivity: 92.38% Specificity: 98.35%	[49]/China
Bovine tuberculosis/*Mycobacterium bovis*	BID109, TB1f, and TB2f	–	MAPIA and Dual Path Platform	125 tuberculosis-positive serum samples/57 healthy bovine serum samples	BD109 Sensitivity: 61.6% Specificity: 94.7% TB1f Sensitivity: 60.8% Specificity: 98.2% TB2f Sensitivity: 77.6% Specificity: 96.5%	[50]/USA
Canine brucellosis/*Brucella* spp.	Multiepitope-based fusion protein	*E. coli* BL21(DE3) cells	Indirect ELISA	34 brucellosis-positive serum samples/62 healthy dog serum samples	Sensitivity: 97.06% Specificity: 100%	[51]/China

“–”: information not provided by the study

Houghton et al. (2002) [39] published the first study with RMPs applied to the diagnosis of bacterial disease. The authors designed TbF6 to diagnose tuberculosis, caused by *Mycobacterium tuberculosis*. To form TbF6, antigens were selected based on the published literature, and, sequentially, *E*. *coli* cells were used for heterologous protein expression. When conducting ELISA assays, TbF6 was combined with a a proline-rich antigen, with sensitivity values ranging from 71.2% to 86% and specificity values from 94.7% to 98.3%. Lin et al. (2008) [40] conducted a study to design a new RMP for the diagnosis of leptospirosis, caused by *Leptospira interrogans*. After applying bioinformatics analyses to the epitope selection, a new RMP, called r-LMP, was obtained using *E*. *coli* BL21(DE3) pLysS cells. Although the sensitivity and specificity values were not provided, results showed that all positive serum samples recognized r-LMP in an ELISA assay. Subsequently, Duthie et al. (2010) [41] developed a new RMP for diagnosing leprosy, caused by *Mycobacterium leprae*. The new RMP, called PADL, was formed by using peptides that reacted with positive serum samples. *E*. *coli* HMS-174 cells were used for PADL expression, and an ELISA assay was performed to verify PADL reactivity with positive human serum samples. Results showed that PADL was recognized by positive samples. However, sensitivity and specificity values were not shown.

Cheng et al. (2011) [42] developed a new RMP that could be used to diagnose human tuberculosis. Epitopes were selected based on published studies to form the fusion protein antigen, which was expressed in *E. coli* BL21(DE3) cells. An ELISA assay was performed to assess antigen reactivity with positive human serum samples, with sensitivity and specificity values of 42.1% and 89.5%, respectively. Later, Li et al. (2015) [43] designed a new RMP, named PstS1-LEP, to diagnose human tuberculosis. After selecting epitopes based on the literature, *E. coli* BL21(DE3) cells were used for PstS1-LEP production. After performing an indirect ELISA assay, results showed that sensitivity values ranged from 42.1% to 82%, depending on the antibody subclass and clinical form of the disease. Specificity values ranged from 72.2% to 95.2%. Yin et al. (2016) [44] conducted a study to construct a new RMP to diagnose human brucellosis, caused by *Brucella* spp. Bioinformatics analyses were performed to select epitopes and *E. coli* BL21(DE3) cells were used to express the new RMP*.* An indirect ELISA assay was performed, with sensitivity and specificity values of 88.89% and 85.54%, respectively.

Schreterova et al. (2017) [45] conducted a study aimed at developing new RMPs for diagnosing Lyme disease, caused by *Borrelia burgdorferi*. After using phage display and multiple alignments for epitope selection, the new RMPs, named A/C-2, A/C-,4, and A/C-7.1, were expressed in *E*. *coli* (XL1-Blue) cells. Among the tested RMPs, A/C-2 and A/C-7.1 showed the best results in the ELISA assay, with 80.17% and 91.37% sensitivity values, respectively. Furthermore, A/C-2 and A/C-7.1 had specificity values of 52.83% and 73.58%, respectively. Next, Yin and colleagues (2020) [46] developed a new RMP, called rOmp, for diagnosing human and goat brucellosis. Epitopes were selected through bioinformatics analyses and *E. coli* BL21(DE3) cells were used for heterologous protein expression. An indirect ELISA was performed to assess rOmp reactivity with positive human and goat serum samples. Results showed that rOmp was able to be recognized by both human and goat positive sera, although sensitivity and specificity values were not determined. Continuing the study, Yin et al. (2021) [47] tested rOmp for goat brucellosis diagnosis. After performing an indirect ELISA, 96.49% sensitivity and 94.44% specificity were obtained.

Yin et al. (2021) [48] developed a new RMP for diagnosing bovine and goat brucellosis. Epitopes were selected after bioinformatics analyses, and *E*. *coli* BL21 cells were used for heterologous protein expression. The multi-epitope fusion protein reactivity was verified through indirect ELISA. Sensitivity and specificity values for bovine brucellosis were determined as 97.85% and 96.61%, respectively, while 98.85% sensitivity and 98.51% specificity were observed using goat serum samples. In a similar approach, Yin et al. (2021) [49] selected epitopes through bioinformatics analyses to form a fusion protein for diagnosing human brucellosis. After performing a nano-p-ELISA assay, sensitivity and specificity values of 92.38% and 98.35, respectively, were determined.

Next, Lyashchenko et al. (2021) [50] developed three new RMPs for diagnosing bovine tuberculosis, caused by *Mycobacterium bovis*. To form these RMPs, named BID109, TB1f, and TB2f, antigens were selected based on the literature. Next, multiantigen print immunoassay and Dual Path Platform (DPP) assay were performed to assess the RMP’s reactivity with positive serum samples. A strong immunoreactivity was observed in the multiantigen print immunoassays for all tested RMPs. As for the DPP results, TB2f showed the best performance, with 77.6% sensitivity and 96.5% specificity. Lastly, Yao et al. (2022) [51] tested the multi-epitope fusion protein’s ability to diagnose canine brucellosis, which was previously designed by Yin et al. (2021) [49]. An indirect ELISA assay was performed, resulting in 97.06% sensitivity and 100% specificity.

### RMPs applied in the diagnosis of diseases caused by fungus

The fungal kingdom has approximately six million species [52, 53], among which 200 have already been described as members of the human microbiome or as human pathogens [54], with 19 of them on the WHO’s fungal priority pathogens list [55]. Moreover, several species are also known to cause animal infections [56]. It is estimated that more than 150 million severe cases and 1.7 million human deaths occur worldwide annually [57]. Despite their importance, fungal diseases have largely been neglected over the years, and reliable diagnoses are only available for a small number of species [58]. Moreover, the diagnostic tests that do exist are not widely available [59, 60]. RMPs has been applied in the experimental diagnoses of infections caused by fungus and Table 2 summarizes the main points of these studies.

**Table 2:** RMPs applied in the diagnosis of diseases caused by fungus

Disease/causative agent	RMP name	Host for protein expression	Serological assay	Samples	Results	Reference/country
Human pneumonia/*Pneumocystis jirovecii*	RSA	*E*. *coli* BL21 Star(DE3) cells	Indirect ELISA	88 pneumonia-positive serum samples/17 serum samples from healthy individuals	Associated with clinical diagnosis-Sensitivity: 100% Specificity: 80.8% Without an associated clinical diagnosis-Sensitivity: 68% Specificity: 61.8%	[61]/Portugal
Human cryptococcosis/*Cryptococcus* spp	Recombinant multiepitope proteins A, B, C, and D	*E. coli* BL21(DE3) cells	ELISA	70 cryptococcosis-positive serum samples/10 serum samples from healthy individuals/68 serum samples from non-cryptococcosis diseases	Protein A Sensitivity: 57.14% Specificity: 90% Protein B Sensitivity: 80% Specificity: 90% Protein C Sensitivity: 88.57% Specificity: 90% Protein D Sensitivity: 88.57% Specificity: 100%	[62]/Brazil
Human pneumonia/*Pneumocystis jirovecii*	Kex1 RSA	*E*. *coli* XJb(DE3) cells	ELISA	48 pneumonia-positive serum samples/104 serum samples from non-*P. jirovecii* infection	Sensitivity: 70.8% Specificity: 75.0%	[63]/Portugal

Keeping in mind the need to develop new diagnostic tests, few studies in the literature have used RMPs in the diagnosis of fungus infections. A study published by Tomás et al. (2016) [61] was the first to use RMPs for this purpose. The authors developed a new RMP, called RSA, to diagnose human pneumonia, caused by *Pneumocystis jirovecii*. The epitopes comprising the new RMP were selected by studying the immunogenicity of a major surface glycoprotein, and RSA was obtained through heterologous expression in *E. coli* BL21 Star(DE3) cells. After performing an in-house ELISA, the diagnosis using RSA presented 100% sensitivity and 80.8% specificity when associated with a clinical diagnosis. When analyzed without associating it with a clinical diagnosis, the sensitivity and specificity values dropped to 68% and 61.8%, respectively.

Brandão et al. (2018) [62] conducted a study to develop a new RMP for the diagnosis of human cryptococcosis, caused by *Cryptococcus* spp. Authors  selected epitopes through bioinformatics analyses to form four new RMPs, called recombinant multiepitope proteins A, B, C, and D. All genes were expressed in *E. coli* BL21(DE3) cells, and an in-house ELISA was used to evaluate their performance. Proteins C and D showed the best results, in which both demonstrated 88.57% sensitivity, and specificity of 90% and 100%, respectively. Lastly, Tomás et al. (2020) [63] developed and tested a new RMP for the diagnosis of human pneumonia, caused by *P. jirovecii*. The epitopes were selected by studying the immunogenicity of *P*. *jirovecii*’s kexin-like serine protease, with the new RMP being designated Kex1 RSA. After obtaining Kex1 RSA using *E*. *coli* XJb(DE3) cells, a sensitivity and specificity of 70.8% and 75.0%, respectively, was confirmed following an indirect ELISA.

### RMPs applied in the diagnosis of diseases caused by protozoa

Protozoa species are found in all possible habitats [64], and although only a small percentage of species are known to be human and animal pathogens [65], they pose an important public health threat with a profound economic impact, being responsible for millions of deaths and significant morbidity worldwide [66]. Millions of human cases are reported annually related to protozoan diseases, such as malaria, leishmaniasis, and Chagas disease. In 2021, an estimated 247 million malaria cases were reported [67]. Moreover, leishmaniasis is responsible for causing nearly one million cases every year [68], and it is currently estimated that six to seven million people worldwide are afflicted with Chagas disease [69]. In view of such a profound economic impact, a rapid, effective, and accessible diagnosis is important for a better prognosis [70], and, in this regard, several studies have focused efforts on the development of new diagnostic tests. RMPs has been applied in the experimental diagnoses of infections caused by protoza and Table 3 summarizes the main points of these studies.

**Table 3:** RMPs applied in the diagnosis of diseases caused by protozoa

Disease/causative agent	RMP name	Host for protein expression	Serological assay	Samples	Results	Reference/country
Human chagas disease/*Trypanosoma cruzi*	ITC 8.2	*E*. *coli* Rosetta2(DE3) pLysS cells	Dipstick assay	118 Chagas-positive serum samples/106 serum samples from non-Chagas diseases	Sensitivity: 99.2% Specificity: 99.1%	[10]/USA
Human chagas disease/*Trypanosoma cruzi*	CP1 and CP2	*E. coli* BL21(DE3) cells	ELISA	141 Chagas-positive serum samples/164 Chagas-negative serum samples/15 cutaneous leishmaniasis-positive serum samples	CP1 was recognized by positive serum samples (sensitivity and specificity values were not shown) CP2 Sensitivity: 98.6% Specificity: 99.4%	[71]/Argentina
Human toxoplasmosis/*Toxoplasma gondii*	rMEP	*E. coli* BL21(DE3) cells	ELISA	108 toxoplasmosis-positive serum samples/42 serum samples from healthy individuals	Sensitivity: 94.4–96.9% Specificity: 96.9%	[72]/China
Human toxoplasmosis/*Toxoplasma gondii*	rMEP	*E*. *coli* BL21(λDE3) cells	ELISA	123 toxoplasmosis-positive serum samples/35 serum samples from healthy individuals	Acute infection Sensitivity: 96.6% Specificity: 100% Past infection Sensitivity: 96.4% Specificity: 98.7%	[30]/China
Human Chagas disease/*Trypanosoma cruzi*	CP2	–	Immunoagglutination assay	16 Chagas-positive serum samples/16 serum samples from healthy individuals	Sensitivity: 92% Specificity: 84%	[73]/Argentina
Canine visceral leishmaniasis/*Leishmania infantum*	PQ10 and PQ20	*E*. *coli* cells	ELISA	231 *L*. *infantum*-positive serum samples/131 healthy dog serum samples	PQ10 Sensitivity: 88.8% Specificity: 80% PQ20 Sensitivity: 84.9% Specificity: 65%	[74]/Brazil
Human toxoplasmosis/*Toxoplasma gondii*	USM.TOXO1	*E. coli* BL21(DE3) pLysS cells	ELISA	40 toxoplamosis-positive serum samples/40 serum samples from healthy individuals	Sensitivity: 100% Specificity: 100%	[31]/Malaysia
Human Chagas disease/*Trypanosoma cruzi*	TcF43 and TcF26	*E*. *coli* cells	ELISA	162 Chagas-positive serum samples/36 serum samples from healthy individuals	TcF43 and TcF26 were recognized by positive serum samples	[75]/USA
Canine visceral leishmaniasis/*Leishmania infantum*	PQ10 and PQ20	*E*. *coli* cells	ELISA	1450 *L*. *infantum*-positive serum samples/42 healthy dogs serum samples	PQ10 and PQ20 were recognized by positive serum samples at earlier time point	[76]/Brazil
Human toxoplasmosis/*Toxoplasma gondii*	USM.TOXO1	*E*. *coli* cells	Indirect ELISA	157 toxoplasmosis-positive serum samples/96 serum samples from healthy individuals/17 serum samples from non-toxoplasmosis diseases	Sensitivity: 85.43% Specificity: 81.25%	[77]/Malasya
Human Chagas disease/*Trypanosoma cruzi*	CP3	*E. coli* BL21(DE3) cells	ELISA	67 Chagas-positive serum samples/67 serum samples from healthy individuals	Sensitivity: 100% Specificity: 90.2%	[78]/Argentina
Canine visceral leishmaniasis/*Leishmania infantum*	PQ10 and PQ20	*E*. *coli* cells	Chemiluminescent ELISA	100 *L*. *infantum*-positive serum samples/30 healthy dog serum samples/32 serum samples from non-*L*. *infantum* diseases	PQ10 Sensitivity: 93.1% Specificity: 80% PQ20 Sensitivity: 93.1% Specificity: 96.6%	[79]/Brazil
Canine visceral leishmaniasis/*Leishmania infantum*	PQ10	*E. coli* BL21(DE3) cells	ELISA/ Direct agglutination	50 *L*. *infantum*-positive serum samples/50 healthy dog serum samples/30 serum samples from non-*L*. *infantum* diseases	ELISA Sensitivity: 94% Specificity: 86% Direct agglutination PQ10 was recognized by 92% of asymptomatic and 96% of symptomatic infected dogs	[80]/Iran
Human toxoplasmosis/*Toxoplasma gondii*	pQE30	*E. coli* BL21(DE3) cells	Indirect ELISA	95 toxoplasmosis-positive serum samples/25 serum samples from healthy individuals/6 serum samples from non-toxoplasmosis diseases	Sensitivity: 72.6% Specificity: 90.3%	[81]/Iran
Pig toxoplasmosis/*Toxoplasma gondii*	MAG	*E. coli* BL21(DE3) cells	ELISA	82 toxoplasmosis-positive serum samples/127 healthy pig serum samples	Sensitivity: 79.1% Specificity: 88.6%	[82]/China
Canine visceral leishmaniasis/*Leishmania infantum*	P1P2P3	*E. coli* BL21(DE3) cells	ELISA	50 *L*. *infantum*-positive serum samples/50 healthy dog serum samples/14 serum samples from non-*L*. *infantum* diseases	Sensitivity: 98% Specificity: 95.31%	[83]/Iran
Human visceral leishmaniasis/*Leishmania infantum*	GRP-UBI-HSP	*E*. *coli* BL21 cells	ELISA	30 *L*. *infantum*-positive serum samples/15 serum samples from healthy individuals	Sensitivity: 70.6% Specificity: 84.1%	[84]/Iran
Human visceral leishmaniasis/*Leishmania infantum*	PQ10	*E. coli* BL21(DE3) cells	ELISA	50 *L*. *infantum*-positive serum samples/50 serum samples from healthy individuals/20 serum samples from non-*L*. *infantum* diseases	Sensitivity: 84% Specificity: 82%	[85]/Iran
Human visceral leishmaniasis/*Leishmania infantum*	MRP	*E. coli* BL21(DE3) cells	ELISA	35 *L*. *infantum*-positive serum samples/20 serum samples from healthy individuals/10 serum samples from non-*L*. *infantum* diseases	Sensitivity: 93.1% Specificity: 77.4%	[86]/Iran
Human and canine visceral leishmaniasis/*Leishmania infantum*	rMELEISH	*E. coli* BL21(DE3) pLysS cells	ELISA	35 human and 45 dog *L*. *infantum*-positive serum samples/30 healthy human and 50 healthy dog serum samples/80 human and 45 dog serum samples from non-*L*. *infantum* diseases	Human and canine diagnosis Sensitivity: 100% Specificity: 100%	[4]/Brazil
Human Chagas disease/*Trypanosoma cruzi*	rTC	*E*. *coli* BL21(λDE3) pLysS cells	ELISA	58 Chagas-positive serum samples/30 serum samples from healthy individuals/60 serum samples from non-Chagas diseases	Sensitivity: 98.28% Specificity: 96.67%	[5]/Brazil

Houghton et al. (2009) [10] published the first study using RMP for the diagnosis of diseases caused by protozoa. The authors developed a new RMP, ITC 8.2, for human Chagas disease diagnosis, caused by *Trypanosoma cruzi.* ITC 8.2 was designed from the combination of another RMP, TcF, with immunodominant peptides. After obtaining ITC 8.2 through expression in *E*. *coli* Rosetta2(DE3) pLysS cells, reactivity with positive human sera sample was analyzed using a dipstick assay. Results showed sensitivity and specificity values of 99.2% and 99.1%, respectively. Also working with Chagas disease diagnosis, Camussone et al. (2009) [71] developed CP1 and CP2 antigens after performing an epitope junction that had shown promising results in an ELISA assay. These RMPs were obtained through heterologous expression using *E. coli* BL21(DE3) cells, and antigenicity was assessed during an ELISA assay. Results showed that CP1 and CP2 presented a greater antigenicity as compared to the mix of peptides that comprised each one. Moreover, CP2 showed better performance, with 98.6% sensitivity and 99.4% specificity.

Later, Dai et al. (2012) [72] worked with a new RMP for the diagnosis of human toxoplasmosis, caused by *Toxoplasma gondii*. Immunodominant epitopes were screened after bioinformatics analyses and selected to form rMEP, which was obtained through expression in *E. coli* BL21(DE3) cells. An ELISA assay was performed to access rMEP reactivity with human positive sera samples. Sensitivity values ranged from 94.4% to 96.9%, depending on the immunoglobulin class, with a specificity value of 100%. In addition, rMEP performance was superior to that of its constituent epitopes when analyzed separately. Continuing the work of the aforementioned study, Dai et al. (2013) [30] evaluated the rMEP’s capacity to differentiate recent from past toxoplasmosis infections. In their study, rMEP was also obtained through expression in *E. coli* BL21(DE3) cells and an in-house ELISA was performed. Results showed that rMEP could be used to differentiate acute from past infection, with sensitivity and specificity values ranging from 96.4% to 96.6% and 98.7% to 100%, respectively.

Garcia et al. (2013) [73] worked with an RMP, named CP2, aimed at diagnosing Chagas disease. This protein had been previously tested, and, in their study, the authors produced a latex-protein complex to be tested in an immunoagglutination assay. Sensitivity and specificity values were determined as 92% and 84%, respectively. In addition, CP2 was more efficient in discriminating between positive and negative serum samples as compared to single recombinant proteins. Next, Faria et al. (2015) [74] developed two new RMPs for the diagnosis of canine visceral leishmaniasis, caused by *Leishmania infantum*. Epitopes were selected based on the literature to form PQ10 and PQ20, and these antigens were obtained after heterologous expression in *E*. *coli* cells. An ELISA assay was performed to assess RMP reactivity with canine sera samples, where sensitivity values were determined as 88.8% and 84.9% for PQ10 and PQ20, respectively. These values were higher as compared to sensitivity values from DPP (Bio-Manguinhos/Fiocruz; Rio de Janeiro, Brazil) and EIE-LVC kit (Bio-Manguinhos/Fiocruz; Rio de Janeiro, Brazil) commercial tests, since sensitivity values for these commercial kits were calculated as 72.9% and 64.5%, respectively. However, PQ10 and PQ20 specificity values were lower compared to commercial tests, showing an 80% and 65% value, respectively, while DPP and EIE-LVC kit showed 90% and 100% specificity, respectively. Next, Hajissa et al. (2015) [31] produced a new RMP for diagnosing human toxoplasmosis infection. The authors selected epitopes based on bioinformatics analyses, and the new RMP antigen, USM.TOXO1, was obtained by heterologous expression in *E. coli* BL21(DE3) pLysS cells. Initially, a Western blotting was performed to verify USM.TOXO1 reactivity with positive serum samples, resulting in recognition by positive human serum samples. The reactivity was further confirmed by ELISA assay, with 100% sensitivity and specificity values.

Duthie et al. (2016) [75] developed two new RMPs, TcF43 and TcF26, for the purpose of diagnosing human Chagas disease. These proteins were expressed in *E*. *coli* cells and evaluated through an ELISA assay. Results showed that TcF43 and TcF26 proteins increased serum recognition as compared to TcF, an antigen used in commercial kits. However, sensitivity and specificity values were not provided. Faria et al. (2017) [76] continued the studies with the RMPs PQ10 and PQ20, previously cited, aimed at diagnosing canine visceral leishmaniasis. After heterologous expression in *E*. *coli* cells, an ELISA assay was performed to assess the antigens’ capacity to detect the disease at early stages. When compared to ELISA based on crude antigens, PQ10, and, especially, PQ20 were able to detect the infection at earlier time points. In addition, these recombinant antigens demonstrated high result concordances in relation to real-time PCR. Hajissa et al. (2017) [77] continued the work developed by Hajissa et al. (2015) [31], employing USM.TOXO1 for diagnosing human toxoplasmosis infection. USM.TOXO1 was obtained using *E*. *coli* cells, and, after an ELISA assay with human sera samples, sensitivity and specificity values were determined as 85.43% and 81.25%, respectively. With the objective of developing a new RMP for diagnosing human Chagas disease, Peverengo et al. (2018) [78] produced CP3 after selecting epitopes based on the published literature. CP3 was obtained through heterologous expression in *E. coli* BL21(DE3) cells, after which an ELISA assay was performed to assess protein antigenicity. Results showed 100% sensitivity and 90.2% specificity values.

In 2019, another study was performed using PQ10 and PQ20 to diagnose canine visceral leishmaniasis. After obtaining the proteins through expression in *E*. *coli* cells, Fonseca et al. (2019) [79] performed a chemiluminescent ELISA to evaluate the antigens’ reactivity with canine serum samples. PQ10 sensitivity and specificity values were determined as 93.1% and 80.0% respectively, while PQ20 showed 93.1% sensitivity and 96.6% specificity. Both PQ10 and PQ20 demonstrated better diagnostic performance as compared to crude antigen diagnostics results. PQ10 was also tested in a study conducted by Jameie et al. (2020) [80]. Following expression in *E. coli* BL21(DE3) cells, an ELISA assay was performed to evaluate protein reactivity with canine visceral leishmaniasis serum samples. Results showed sensitivity and specificity values of 94% and 86%, respectively. Moreover, a direct agglutination test assay was performed, in which PQ10 was able to detect 92% of asymptomatic and 96% of symptomatic infected dogs.

Alibakhshi et al. (2020) [81] developed a new RMP, pQE30, for diagnosing human toxoplasmosis. To construct pQE30, epitopes were selected using bioinformatics analyses and *E. coli* BL21(DE3) cells were used to obtain the recombinant antigen. An ELISA assay was then performed, with sensitivity and specificity values of 72.6% and 90.3%, respectively. While conducting a study aimed at diagnosing animal toxoplasmosis, caused by *T*. *gondii*, Song et al. (2021) [82] selected epitopes after performing bioinformatics analyses to form a new RMP, called MAG, which was expressed in *E. coli* BL21(DE3) cells. A Western blotting assay was then performed, with positive pig sera samples recognizing MAG. Reactivity was further confirmed by an ELISA assay, showing 79.1% sensitivity and 88.6% specificity.

Yaghoubi et al.. (2021) [83] conducted a study to construct a new RMP for canine visceral leishmaniasis diagnosis. Bioinformatics analyses were used to select epitopes for the new RMP development, named P1P2P3. This antigen was obtained after expression in *E. coli* BL21(DE3) cells, and an ELISA assay was performed to access antigen reactivity. Results showed a 98% sensitivity and 95.31% specificity, demonstrating agreement with the direct agglutination test, the gold standard test used in the study. Working with human visceral leishmaniasis, caused by *L*. *infantum*, Heidari et al. (2021) [84] developed a new RMP, called GRP-UBI-HSP, to be tested in ELISA assay. In their study, epitopes were selected from immunoreactive proteins through bioinformatic analyses, and GRP-UBI-HSP was obtained after expression in *E*. *coli* BL21 cells. The results of an ELISA assay showed 70.6% sensitivity and 84.1% specificity. A subsequent study, performed by Jameie et al. (2021) [85], aimed to verify PQ10’s diagnostic capacity for human visceral leishmaniasis, caused by *L*. *infantum*. PQ10 had already been tested in several studies for the diagnosis of canine visceral leishmaniasis, with promising results. In their study, the antigen was obtained through heterologous expression in *E. coli* BL21(DE3) cells, and an ELISA assay was performed to evaluate PQ10 antigenicity. Results showed a diagnostic performance of 84% sensitivity and 82% specificity.

Subsequently, Taherzadeh et al. (2021) [86] developed a new RMP for human visceral leishmaniasis diagnosis caused by *L*. *infantum*. Epitopes were selected based on bioinformatics analyses, and, after designing the recombinant antigen named MRP, the heterologous antigen was expressed using *E. coli* BL21(DE3) cells. Initially, MRP recognition by human positive serum samples was confirmed through Western blotting analyses. Results derived from an ELISA assay demonstrated 93.1% sensitivity and 77.4% specificity. Next, Dias et al. (2023) [4] conducted a study to evaluate the diagnostic efficiency of a new RMP, rMELEISH, for both human and canine visceral leishmaniasis, caused by *L*. *infantum*. After selecting epitopes based on the literature, rMELEISH was obtained through expression in *E. coli* BL21(DE3) pLysS cells and ELISA assays showed 100% sensitivity and specificity values. The same results were observed for canine sera sample reactivity. Moreover, rMELEISH demonstrated a better diagnostic performance as compared to results using soluble *Leishmania* antigen extract. Lastly, a novel RMP for diagnosing Chagas disease was developed by Machado et al. (2023) [5]. To compose rTC antigen, the epitopes were selected based on the literature. *E. coli* BL21(DE3) pLysS cells were used to obtain rTC, and an ELISA assay was performed. Results showed sensitivity and specificity values of 98.28% and 96.67%, respectively.

### RMPs applied in the diagnosis of viral diseases

Among the species, 219 viral species are recognized as human pathogens [87, 88], and several species also affect animals [89, 90]. Viral infections represent a major public health problem and humanity has faced several lethal viral pandemics, such as Spanish flu and COVID-19, causing the death of millions of people worldwide [91]. Moreover, viral infections also cause thousands of deaths annually without being related to endemics or pandemics [92, 93]. Given its importance, much effort has been applied to the development of viral disease diagnoses, which are currently performed through several methods. Among them, the serological method is widely applied due to its high sensitivity and specificity, low cost, and rapid diagnosis [94, 95]. RMPs has been applied in the experimental diagnoses of infections caused by virus and Table 4 summarizes the main points of these studies.

**Table 4:** RMPs applied in the diagnosis of viral diseases

Disease/causative agent	RMP name	Host for protein expression	Serological assay	Samples	Results	Reference/country
Human dengue fever/dengue virus	r-DME-G	*E*. *coli* strain SG13009 cells	ELISA	10 dengue-positive serum samples/10 serum samples from healthy individuals	Sensitivity: 100% Specificity: not provided	[9]/India
Human dengue fever/dengue virus	r-DME-M	*E*. *coli* cells	ELISA	22 dengue-positive serum samples/150 dengue-negative serum samples	r-DME-M was recognized by all positive human samples	[96]/India
Human hepatitis C/hepatitis C virus	r-HCV-F-MEP	*E. coli* BL21(DE3) cells	ELISA/Lateral flow assay	> 200 hepatitis-positive serum samples/100 serum samples from healthy individuals	ELISA Sensitivity: 99.8% Specificity: 100% Lateral flow 100% compatible with ELISA results	[1]/India
Human dengue fever/dengue virus	rDME-M	*E*. *coli* cells	Dipstick ELISA	81 dengue-positive serum samples/39 serum samples from healthy individuals/30 serum samples from non-dengue diseases	Sensitivity: 100% Specificity: 85% and 93%	[97]/India
Human dengue fever/dengue virus	rDME-G	*E*. *coli* cells	Dipstick ELISA	50 dengue-positive serum samples/10 serum samples from healthy individuals	Sensitivity: 100% and 95% Specificity: 100%	[98]/India
Human HIV/human immunodeficincy virus	HIV-MEP	*E. coli* BL21(DE3) cells	Indirect ELISA	57 HIV-positive serum samples/50 HIV-negative serum samples	High sensitivity and specificity values were observed	[99]/India
Human hepatitis C/hepatitis C virus	HCV	*E*. *coli* cells	Double-antigen sandwich ELISA	259 hepatitis-positive serum samples/440 serum samples from healthy individuals/190 serum samples from non-hepatits C diseases	Sensitivity: 98.7% Specificity: 100%	[100]/China
Human hepatitis C/hepatitis C virus	rHCV-MEP	*E. coli* BL21(DE3) cells	ELISA	56 hepatitis-positive serum samples/50 hepatitis C-negative serum samples	High sensitivity and specificity values were observed	[101]/India
Human hepatitis B/hepatitis B virus	rMEHB	*E. coli* BL21(DE3) pLysS cells	ELISA	–	rMEHB was recognized by human positive samples	[19]/Brazil
EBV-associated tumors/epstein-barr virus	EBV-LMP2m	*E. coli* BL21(DE3) cells	ELISA	238 Epstein-Barr-positive serum samples/112 serum samples from healthy individuals	Sensitivity: 52.84% Specificity: 95.40%	[102]/China
Human hepatitis A/hepatitis A virus	H1	*E. coli* BL21(DE3) cells	Double-antigen sandwich ELISA	48 hepatitis-positive serum samples/96 serum samples from vaccinated individuals	Sensitivity: 93.75% Specificity: 93.75%	[103]/China
Human hepatitis C/hepatitis C virus	rMEHCV	*E*. *coli* BL21(λDE3) pLysS cells	ELISA	17 hepatitis-positive serum samples/10 serum samples from healthy individuals	High sensitivity and specificity values were observed	[2]/Brazil
Human hepatitis C/hepatitis C virus	r-HCV-MEPs	*E. coli* BL21(DE3) cells	Secondary antibody/ Double-antigen immunoassays	113 hepatitis-positive serum samples/58 hepatitis-negative serum samples	Secondary antibody Sensitivity: 95.6% Specificity: 100% Double-antigen assay Sensitivity: 91.4% Specificity: 100%	[104]/Finland
Swine foot-and-mouth disease/foot-and-mouth disease virus	B4	*E*. *coli* cells	Indirect ELISA	147 foot-and-mouth-positive serum samples/610 healthy swine serum samples	Sensitivity: 95.9% Specificity: 96.7%	[105]/China
Human hepatitis C/hepatitis C virus	Recombinant multiepitope HCV antigen	*E*. *coli* cells	ELISA	88 hepatitis-positive serum samples/376 hepatitis C-negative serum samples/18 serum samples from non-hepatitis C diseases	Sensitivity: 100% Specificity: 99.73%	[106]/Brazil
Human cytomegalovirus/cytomegalovirus	rMEHCMV	*E. coli* BL21(DE3) cells	ELISA	12 cytomegalovirus-positive serum samples/1 serum sample from healthy individual	rMEHCMV was recognized by human positive samples	[107]/Brazil
Canine coronavirus/SARS-CoV-2	rSP	*E. coli* BL21(DE3) cells	Indirect ELISA	64 coronavirus-positive serum samples/10 healthy dog serum samples	82.81% positive rate	[108]/China
Human coronavirus/SARS-CoV-2	Dx-SARS2-RBD and Dx-SARS2-noRBD	*E. coli* BL21(DE3) cells	ELISA	185 coronavirus-positive serum samples/256 serum samples from healthy individuals/94 serum samples from non-coronovirus diseases	Dx-SARS2-RBD Sensitivity: 100% Specificity: 99.51-100% Dx-SARS2-noRBD Sensitivity: 100% Specificity: 99.21-100%	[109]/Brazil
African swine fever/African swine fever virus	reMeP72	*E. coli* BL21(DE3) cells	Colloidal gold-based immunochromatographic assay	139 swine serum samples	Sensitivity: 85.7% Specificity: 97.6%	[110]/China
African swine fever/African swine fever virus	m35	*E*. *coli* BL21 cells	Indirect ELISA	78 positive serum samples/215 negative serum samples	Sensitivity: 98.72% Specificity: 98.14%	[111]/China
Human mayaro fever/mayaro virus	Dx-MAYV-M	*E. coli* BL21(DE3) cells	ELISA	18 mayaro-positive serum samples/40 serum samples from healthy individuals	Sensitivity: 99.6% Specificity: 100%	[6]/Brazil
Swine foot-and-mouth/foot-and-mouth disease virus	ME protein	*E. coli* BL21(DE3) cells	Indirect chemiluminescence immunoassay	118 foot-and-mouth-positive serum samples/307 healthy swine serum samples	Sensitivity: 100% Specificity: 99.35%	[112]/China
Human hepatitis C/hepatitis C virus	MBP-rHCV	*E. coli* BL21(DE3) cells	Indirect ELISA	142 hepatitis-positive serum samples/172 serum samples from healthy individuals	Sensitivity: 95% Specificity: 92%	[113]/Canada
Human rubella/rubella virus	rMERUB	*E*. *coli* BL21(λDE3) cells	ELISA	22 rubella-positive serum samples/11 rubella-negative serum samples	Sensitivity: 100% Specificity: 90.91%	[114]/Brazil
Human HTLV-1 and HTLV-2/human T-lymphotropic viruses 1 and 2	HTLV-1/HTLV-2	*E*. *coli* Rosetta-gami 2(DE3) cells	ELISA	162 HTLV-positive serum samples/297 serum samples from healthy individuals/92 serum samples from non-HTLV disease	HTLV-1 and HTLV-2 samples Sensitivity: 82.41 to 92.36% Specificity: 90.09% to 95.19% HTLV-1 samples Sensitivity: 99.19% Specificity: 92.55% HTLV-2 samples Sensitivity: 57.14% Specificity: 94.61%	[115]/Brazil
Human chikungunya/chikungunya virus	MULTREC	Binary system insect cell/baculovirus	ELISA	161 chikungunya-positive serum samples/22 serum samples from healthy individuals/312 serum sample from non-chikungunya diseases	Sensitivity: 86.36% Specificity: 100%	[116]/Brazil

“–”: information not provided by the study

Ananda Rao et al. (2005) [9] published the first study describing the use of an RMP for the diagnosis of a human viral infection. In their study, the epitopes were selected through phage display, pepscan, and computer analysis of three selected proteins of the dengue virus, responsible for causing dengue fever. The epitopes were combined to form a new RMP, identified as r-DME-G, which was obtained in *E*. *coli* strain SG13009 cells. An in-house ELISA was performed, resulting in 100% sensitivity. Specificity data were not provided. Next, Ananda Rao et al. (2006) [96] designed a new RMP, identified as r-DME-M, which was also applied to human dengue infection diagnosis. The epitopes were selected using the same approach as described in the 2005 study, and r-DME-M was obtained using *E*. *coli* cells. After performing an in-house ELISA, it was observed that r-DME-M was capable of detecting all seropositive human samples, demonstrating better performance than the commercial test PanBio (Pty Ltd.; Windsor, Australia), used for comparison. However, sensitivity and specificity values were not available.

Subsequently, Dipti et al. (2006) [1] developed a new RMP for human hepatitis C diagnosis. The epitopes were selected based on the literature, forming a new RMP, r-HCV-F-MEP, which was expressed in *E. coli* BL21(DE3) cells. The in-house ELISA showed a 99.8% sensitivity and 100% specificity. Moreover, a lateral flow assay was performed, and the results were fully compatible with those obtained in the in-house ELISA. Next, Tripathi et al. (2007) [97] developed a new RMP for human dengue diagnosis, identified as rDME-M, obtained through expression in *E*. *coli* cells. Initially, rDME-M reactivity was verified by Western blotting, in which a seropositive human sample recognized rDME-M. An in-house dipstick ELISA was then performed, with 93% and 85% specificity as compared to reference available ELISA and rapid immunochromatography tests, respectively. Moreover, the sensitivity of the in-house dipstick ELISA was calculated as 100% as compared to both reference tests. Tripathi et al. (2007) [98] also worked with a new RMP for diagnosing human dengue infection. In this study, the epitopes were selected based on phage display and computer predictions to form a new RMP, identified as rDME-G. After analyzing the reactivity of rDME-G with human sera in an in-house dipstick ELISA, the sensitivity was 100% and 95% as compared to reference available ELISA and rapid immunochromatography tests, respectively. Moreover, the specificity value was 100% as compared to both reference tests.

Talha et al. (2010) [99] developed HIV-MEP for the diagnosis of HIV infection. In their study, the authors selected the epitopes based on the literature, and a new RMP formed was obtained after expression in *E*. *coli* strain BL21 (DE3) cells. To assess HIV-MEP reactivity, an in-house indirect immunoassay was performed, and the results were similar to those obtained using Abbott HIV-1, Abbott HIV-1/2, Genetic Systems HIV-1, Genetic Systems HIV-1/2, and Organon Teknika HIV-1 commercial tests. Despite having high sensitivity and specificity, their values were not provided. Next, He et al. (2011) [100] constructed a new RMP for diagnosing human hepatitis C. After obtaining the new recombinant multiepitope HCV antigen through expression in *E*. *coli* cells, a double-antigen sandwich ELISA was performed to verify antigen reactivity. The results were similar to those obtained with Ortho ELISA 3.0 (GWK; Beijing, China) commercial test, where the sensitivity and specificity of both developed tests and commercial kit were 98.8% and 100%, respectively. Also working with the diagnosis of hepatitis C, Gurramkonda et al. (2012) [101] developed a new RMP, designated as rHCV-MEP. To compose this new RMP, epitopes were selected based on the literature, and rHCV-MEP was obtained using *E. coli* BL21(DE3) cells. An in-house ELISA assay was applied to evaluate performance, and a high sensitivity and specificity was observed, with results compatible with those obtained using Abbott HCV 2.0, Abbott HCV 3.0, Ortho HCV 2.0, and Ortho HCV 3.0 commercial tests. However, sensitivity and specificity values were not provided.

Subsequently, de Souza et al. (2013) [19] worked with a new RMP, identified as rMEHB, for the diagnosis of human hepatitis B. Conserved epitopes were selected and *E. coli* BL21(DE3) pLysS cells were used to obtain it. In their study, the TIEBK PLUS No 140—Diasorin commercial kit was used, with the commercial antigen being replaced by rMEHB. The results showed that rMEHB was recognized by antibodies in human positive samples and its performance was similar to that of the commercial test. Next, Lin et al. (2016) [102] developed a new RMP for the diagnosis of human Epstein-Barr virus-associated tumors. For epitope selection, bioinformatics analyses were conducted, and the new antigen was named EBV-LMP2m. Western blotting was used to verify EBV-LMP2m reactivity and confirm recognition by positive human serum. Moreover, the EBV-LMP2m performance was evaluated in an in-house ELISA, resulting in 52.84% sensitivity and 95.40% specificity. Su et al. (2016) [103] designed a new RMP aimed at diagnosing human hepatitis A. To form this new RMP, identified as H1, the authors selected immune-dominant epitopes based on previous studies. After obtaining H1 through expression in *E*. *coli* BL21(DE3), a double-antigen sandwich ELISA was performed to assess serum-RMP reactivity, with sensitivity and specificity values of 93.75%.

Galdino et al. (2016) [2] developed a new RMP for the diagnosis of human hepatitis C. In that study, the epitopes were selected based on the literature and the new RMP, rMEHCV, was obtained through expression in *E. coli* BL21(DE3) pLysS cells. Results of an in-house ELISA showed 100% agreement with those of the Hepanóstika HCV Ultra^®^ (Beijing, China) commercial test, with high sensitivity and specificity values. However, these values were not provided. Also working with hepatitis C diagnosis, Salminen et al. (2016) [104] developed the antigen named r-HCV-MEPs, after epitope selection based on the literature. *E. coli* BL21(DE3) cells were used for heterologous protein expression. To assess the r-HCV-MEPs reactivity with positive serum samples, a secondary antibody and double-antigen immunoassays were used, resulting in sensitivity values of 95.6% and 91.4%, respectively. Moreover, specificity values were 100% in both immunoassays. Cao et al. (2018) [105] worked with a RMP, designated as B4, for the diagnosis of animal foot-and-mouth disease. B4 had already been developed in a previous study. After expression in *E*. *coli* cells, an indirect ELISA was performed using swine serum samples, and the results showed 95.9% sensitivity and 96.7% specificity values.

Subsequently, Thomasini et al. (2018) [106] developed a new RMP for diagnosing human hepatitis C. The epitopes were selected based on previously published studies and the new RMP was obtained after expression in *E*. *coli* cells. Initially, an immunoblot assay was performed to assess RMP reactivity, where a strong reaction with positive human samples was observed. After performing an ELISA assay, sensitivity and specificity values were defined as 100% and 99.73%, respectively. Later, Ribeiro et al. (2019) [107] worked with a new RMP, rMEHCMV, for the diagnosis of human cytomegalovirus infection. The authors selected conserved epitopes to form rMEHCMV, and *E. coli* BL21(DE3) cells were chosen for the heterologous expression. An in-house ELISA assay was performed, and results showed that rMEHCMV was recognized by human-infected samples, demonstrating a stronger reactivity as compared to those of the ETI-CYTOK-G PLUS (DiaSorin; Saluggia, Italy) commercial kit. However, sensitivity and specificity values were not provided. Hao et al. (2021) [108] developed a new RMP, rSP, aimed at diagnosing canine coronavirus diagnosis, caused by SARS-CoV-2. Epitopes were selected through bioinformatics analyses, and, after designing the new RMP, *E. coli* BL21(DE3) cells were used for heterologous protein expression. An indirect ELISA assay was performed, where the authors observed good sensitivity and specificity results. However, those values were not provided.

Also working with SARS-CoV-2 diagnosis, Gomes et al. (2021) [109] developed two new RMPs for the diagnosis of human coronavirus. Epitope selection was made through direct microsynthesis of phosphopeptides on membranes synthesis to form Dx-SARS2-RBD and Dx-SARS2-noRBD and both antigens were expressed using *E. coli* BL21(DE3) cells. An in-house ELISA assay was performed to assess reactivity and the results showed 100% sensitivity for both RMPs. Specificity values ranged from 99.51–100% for Dx-SARS2-RBD and 99.21–100% for Dx-SARS2-noRBD. Zhang et al. (2021) [110] worked with a new RMP for the diagnosis of African swine fever. The new antigen, designated reMeP72, was constructed based on epitopes selected by bioinformatics analyses. After obtaining the new antigen using *E. coli* BL21(DE3) cells, a colloidal gold-based immunochromatographic assay was performed. Results indicated 85.7% sensitivity and 97.6% specificity, showing an agreement rate of 96.4% with the ASFV indirect ELISA kit (INGENASA; Madrid, Spain) commercial kit. Also working with the diagnosis of African swine fever in animals, Gao et al. (2021) [111] developed a new RMP, m35, after selecting epitopes through bioinformatics analyses. The m35 protein was obtained after expression in *E*. *coli* BL21 cells, and an indirect ELISA assay was performed to verify reactivity with swine serum samples. Sensitivity and specificity values were defined as 98.72% and 98.14%, respectively.

In the same year, Napoleão‑Pêgo et al. (2021) [6] developed Dx-MAYV-M, a new RMP to be tested in human Mayaro fever diagnosis. After epitope mapping, selected epitopes were joined together and the recently formed Dx-MAYV-M was obtained using *E. coli* BL21(DE3) cells. Initially, Western blotting was performed to verify reactivity, in which antigen recognition by positive human sera was observed. An in-house ELISA assay was performed, resulting in estimated sensitivity and specificity values of 99.6% and 100%, respectively. Next, a new RMP for the diagnosis of animal foot-and-mouth disease was developed by Liu et al. (2021) [112]. In their work, the authors expressed this new RMP, identified as ME protein, using *E*. *coli* BL21(DE3) cells, and performed an indirect chemiluminescence immunoassay to evaluate ME reactivity with swine serum samples. Results showed 100% sensitivity and 99.35% specificity. Later, Pedersen et al. (2022) [113] designed a new RMP for hepatitis C diagnosis, labeled MBP-rHCV. To construct this protein, epitopes were selected based on published studies and *E. coli* BL21(DE3) cells were used for heterologous expression. An indirect ELISA assay was performed to determine MBP-rHCV reactivity with human sera samples, resulting in 95% sensitivity and 92% specificity.

Souza et al. (2022) [114] created a new RMP, named rMERUB, for the diagnosis of human rubella. Conserved epitopes were selected to construct rMERUB, which was obtained through expression in *E. coli* BL21(DE3) cells. After performing an in-house ELISA assay, sensitivity and specificity values were defined as 100% and 90.91%, respectively. Franco et al. (2022) [115] worked with a new RMP for human HTLV-1 and HTLV-2 infections diagnosis, caused by human T-lymphotropic viruses 1 and 2. The HTLV-1/HTLV-2 multiepitope protein was constructed based on previous studies and obtained through expression in *E*. *coli* Rosetta-gami 2 (DE3) cells. A Western blot was then performed, confirming multiepitope protein recognition by positive HTV-1 and HTV-2 serum samples. After an in-house ELISA assay, sensitivity and specificity values ranged from 82.41 to 92.36% and 90.09 to 95.19%, respectively, when considering positive samples for both HTLV1- and HTLV-2. When considering only HTLV-1 samples, sensitivity and specificity values were calculated as 99.19% and 92.55%, respectively. Considering the values when evaluating only HTLV-2 samples, sensitivity and specificity were determined as 57.14% and 94.61%, respectively. Lastly, da Silva et al. (2022) [116] developed a new RMP for diagnosing human chikungunya, caused by the chikungunya virus. This new RMP, MULTREC, was obtained through a binary system insect cell/baculovirus, after which an ELISA assay was performed to assess protein reactivity, with 86.36% sensitivity and 100% specificity.

### RMPs applied in the diagnosis of diseases caused by worms

Infections caused by worms, also known as helminths, are one the most common diseases in the world, with estimates of approximately 1.5 billion people infected worldwide [117]. This group of diseases mainly affects people living in the world’s poorest countries and is associated with severe morbidity [118]. However, despite of the risk that these diseases pose to human and animal lives, they remain poorly studied compared to other disease groups [119]. If this scenario is to change, more efforts must be applied in scientific research, including the development of new diagnostic kits for helminths [120]. RMPs has been applied in the experimental diagnoses of infections caused by worms and Table 5 summarizes the main points of these studies.

**Table 5:** RMPs applied in the diagnosis of diseases caused by worms

Disease/causative agent	RMP name	Host for protein expression	Serological assay	Samples	Results	Reference/country
Goat schistosomiasis/*Schistosoma japonicum*	rBSjPGM-BSjRAD23-1-BSj23, rBSjRAD23-2-BSjPGM-BSj23, rBSjPGM-BSj23, and rBSjPGM-BSjRAD23-1	*E*. *coli* cells	ELISA	91 schistosomiasis-positive serum samples/44 healthy goat serum samples/49 serum samples from non-schistosomiasis diseases	rBSjPGM-BSjRAD23-1-BSj23 Sensitivity: 97.8% Specificity: 100% rBSjRAD23-2-BSjPGM-BSj23 Sensitivity: 89.01% Specificity: 100% rBSjPGM-BSj23 Sensitivity: 93.41% Specificity: 100% rBSjPGM-BSjRAD23-1 Sensitivity: 59.34% Specificity: 97.73%	[32]/China
Buffalo schistosomiasis/*Schistosoma japonicum*	rBSjPGM-BSjRAD23-1-BSj23 and rBSjRAD23-2-BSjPGM-BSj23	*E*. *coli* cells	ELISA	114 schistosomiasis-positive serum samples/92 healthy buffalo serum samples/14 serum samples from non-schistosomiasis diseases	rBSjPGM-BSjRAD23-1-BSj23 Sensitivity: 95.61% Specificity: 97.83% rBSjRAD23-2-BSjPGM-BSj23 Sensitivity: 67.54% Specificity: 100%	[121]/China
Bovine cysticercosis/*Taenia saginata*	rqTSA-25	*E*. *coli* BL21-Codon-Plus(DE3)-RIL cells	ELISA/immunoblot assay	60 cysticercosis-positive serum samples/30 healthy bovine serum samples/15 serum samples from non-cysticercosis diseases	ELISA Sensitivity: 93.3% Specificity: 95.3% Immunoblot no false positive or false negative was observed	[122]/Brazil
Sheep cystic echinococcosis/*Echinococcus granulosus*	reEg mefAg-1	*E. coli* BL21(DE3) cells	Indirect ELISA	86 echinococcosis-positive serum samples/30 echinococcosis-negative serum samples/21 serum samples from non- *Echinococcus granulosus* diseases	Sensitivity: 93.41% Specificity: 99.31%	[123]/China
Human onchocerciasis/*Onchocerca volvulus*	OvNMP-48	–	ELISA	101 onchocerciasis-positive serum samples/58 serum samples from healthy individuals/54 serum samples from non-onchocerciasis diseases	Sensitivity: 76.0% Specificity: 97.4%	[124]/Belgium
Human fascioliasis/*Fasciola hepatica*	rMEP	*E*. *coli* BL21 cells	Western blot	–	rMEP was recognized by human positive samples	[125]/Iran
Human lymphatic filariasis/*Wuchereria bancrofti*	Multiepitope antigen	*E*. *coli* Rosetta cells	Indirect ELISA	70 lymphatic filariasis-positive serum samples/176 serum samples from healthy individuals/18 serum samples from non-lymphatic filariasis diseases	Sensitivity: 100% Specificity: 98.1% and 99.52%	[126]/India
Human cystic echinococcosis/*Echinococcus granulosus*	rMEP	*E. coli BL21(DE3)* cells	ELISA	43 echinococcosis-positive serum samples/120 serum samples from healthy individuals/23 serum samples from non-echinococcosis diseases	Sensitivity: 95.3% Specificity: 95.0%	[127]/Iran
Human cystic echinococcosis/*Echinococcus granulosus*	DIPOL	*E*. *coli* cells	ELISA	149 echinococcosis-positive serum samples/21 serum samples from healthy individuals/49 serum samples from non-echinococcosis diseases	Sensitivity: 75.4% for active and transitional cysts and 95.6% for inactive cysts Specificity: 97.71%	[128]/Turkey
Human onchocerciasis/*Onchocerca volvulus*	OvMCBL02	–	Indirect ELISA	63 onchocerciasis-positive serum samples/55 serum sample from healthy individuals/21 serum samples from non-onchocerciasis diseases / 54 onchocerciasis-negative serum samples from ivermectin treated individuals	Sensitivity: 98.4% Specificity: 100%	[129]/Belgium

“–”: information not provided by the study

Despite its importance, RMP application in the diagnosis of diseases caused by worms is recent**.** Lv et al. (2016) [32] evaluated the performance of four RMP molecules for diagnosing goat schistosomiasis, caused by *Schistosoma japonicum*. These new RMPs were designed using epitopes selected through bioinformatics analyses, and *E*. *coli* cells were used for heterologous expression. RMPs showed greater sensitivity as compared to single molecular recombinant antigens in ELISA assays, with emphasis on the rBSjPGM-BSjRAD23-1-BSj23 antigen, which showed 97.8% sensitivity and 100% specificity, as compared to soluble egg antigen. Continuing the studies with the RMPs cited above, Lv et al. (2018) [121] evaluated the diagnostic capacity of two RMPs for buffalo schistosomiasis caused by *S*. *japonicum*. Similar to the findings in the previous study, the rBSjPGM-BSjRAD23-1-BSj23 antigen showed the best performance in an ELISA assay, with sensitivity and specificity rates of 95.61% and 97.83%, respectively, again as compared to the soluble egg antigen results. Next, Guimarães-Peixoto et al. (2018) [122] conducted a study to develop a new RMP for diagnosing bovine cysticercosis, caused by *Taenia saginata*. For this purpose, bioinformatics analyses were used to select epitopes, and the new RMP, identified as rqTSA-25, was produced in *E*. *coli* BL21-Codon-Plus(DE3)-RIL cells. After an ELISA assay, rqTSA-25 showed 93.3% sensitivity and 95.3% specificity values. Furthermore, no false positive or false negative reaction was observed in the samples analyzed by the immunoblot test.

Tianli et al. (2019) [123] developed a new RMP for diagnosing sheep cystic echinococcosis, caused by *Echinococcus granulosus*. After conducting bioinformatics analyses for selecting epitopes, a new RMP was designed, named reEg mefAg-1. *E*. *coli* BL21(DE3) cells were used for antigen production, and an indirect ELISA assay was performed to assess reEg mefAg-1’s reactivity. Results showed 93.41% sensitivity and 99.31% specificity. Moreover, reEg mefAg-1-based ELISA results were similar to those found in the IgG ELISA Kit (ab108733, Abcam; Cambridge, Massachusetts, USA) commercial kit. In that same year, Lagatie et al. (2019) [124] conducted a study aimed at diagnosing human onchocerciasis, caused by *Onchocerca volvulus*. For this purpose, a new RMP, OvNMP-48, was constructed based on epitopes selected through proteome-wide screen as performed in previous studies. After an ELISA assay, sensitivity and specificity values were determined as 76.0% and 97.4%, respectively. Moreover, OvNMP-48-ELISA showed greater sensitivity values as compared to epitope-based ELISA. However, an OvNMP-48-based ELISA assay also demonstrated an increase in cross-reactions. Subsequently, Aghamolaei et al. (2020) [125] developed a new RMP for diagnosing human fascioliasis, caused by *Fasciola hepatica*. In their study, bioinformatics analyses were applied to select epitopes and a new RMP, designated as rMEP, was obtained after heterologous expression in *E*. *coli* BL21 cells. A Western blot was performed to assess rMEP’s reactivity with positive human serum samples. A strong band was observed using human positive serum samples, but no band was observed in poled serum with other helminths and healthy individuals. Despite the promising results, no further serological tests were performed.

Yasin et al. (2020) [126] conducted a study to develop a new RMP for diagnosing human lymphatic filariasis, caused by *Wuchereria bancrofti*. To form the new RMP, identified as an multiepitope antigen, epitopes were selected based on previous bioinformatics studies. The multiepitope antigen was obtained using *E*. *coli* Rosetta cells, and an ELISA assay was performed to assess the antigen’s reactivity with human-positive serum samples. Results showed 100% sensitivity, and a specificity range from 98.1 to 99.52%, depending on the antibody subclass detected. Mirzapour et al. (2020) [127] developed a new RMP aimed at diagnosing human cystic echinococcosis, caused by *Echinococcus granulosus*. Epitopes were selected through bioinformatics analyses, and, after designing the new RMP, named rMEP, *E. coli* BL21(DE3) cells were used for heterologous protein expression. ELISA assay results showed high sensitivity and specificity values, determined as 95.3% and 95.0%, respectively. However, the Euroimmun commercial kit showed better performance, with 100% for both sensitivity and specificity. More recently, Ozturk et al. (2022) [128], also working on diagnosing human cystic echinococcosis, tested an RMP, named as DIPOL. In their study, the authors obtained DIPOL through expression in *E*. *coli* cells and performed an ELISA assay to verify antigen reactivity. The DIPOL-based ELISA test showed sensitivity values of 75.4% for active and transitional cysts and 95.6% for inactive cysts. Moreover, specificity values were determined as 97.71%. Lastly, Yengo et al. (2022) [129] developed a new RMP for diagnosing human onchocerciasis. Epitopes were selected through bioinformatics analyses to form the new RMP, OvMCBL02. After performing an indirect ELISA assay, sensitivity and specificity values were determined as 98.4% and 100%, respectively.

## *Escherichia coli*: the platform of choice for RMP production

Although various host cells described in the literature can serve as expression systems for recombinant protein production, almost all diagnostic RMP studies to date have used *E. coli* as the expression system of choice (Tables 1–5). What makes this host so well-suited for this purpose? This expression system boasts a long history and offers several well-established advantages, including ease of manipulation, low-cost culture, and rapid growth kinetics. The doubling time of 20 min facilitates the achievement of high cell density cultures, with a theoretical concentration limit of ~ 1 × 10^13^ viable bacteria/mL [26, 130]. Furthermore, *E. coli* stands out as the most cost-effective host, allowing for the attainment of high cellular densities with inexpensive culture media. Additionally, well-developed tools for molecular manipulations, coupled with in-depth knowledge of its biology [131], contribute to the versatility of bacterium as a protein expression host.

The *E. coli* cultivation process involves growing the bacteria in a culture medium, with selection antibiotics, until reaching an optical density_600_ (OD)_600_ between 0.6 and 0.8, a mid-log phase indicative. For that, the most widely used media are Luria–Bertani, Terrific Broth, and Super Broth, based on mixtures of tryptone/peptone and yeast extract in a saline solution, which can either be sodium chloride or sodium phosphate. To optimize growth, the bacterial culture must be kept at a temperature and rotation of 37 °C and 150–200 revolutions per minute (rpm). Upon reaching the desired absorbance, the inducer molecule must be added to the culture medium, typically the allolactose analog isopropyl β-D-1-thiogalactopyranose (IPTG), to start recombinant gene transcription. More details of this approach are well provided by Sambrook et al. (1989) [132].

The first *E. coli* isolate was deposited in the National Collection of Type Cultures (NCTC, UK) in 1920. Later on, Cohen et al. (1973) made a groundbreaking discovery of recombinant DNA technology, which marked a pivotal moment in the biotechnology field five decades ago [133]. Successful productions of human somatostatin [134] and insulin [135] were quickly achieved in those cells. From then on, its ease of genetic manipulation has allowed for the insertion and knockout of genes, resulting in several strains better suited for each recombinant protein profile. Although there is a clear preference for *E. coli* B derivative strains in RMP surveys (BL21 and BL21(DE3)), there are other useful lineages as well. For example, there are strains more effective in preventing the formation of inclusion bodies, a challenge frequently discussed in the literature concerning *E. coli* in protein production. The ideal strain to use will depend on the specific requirements of the protein being expressed.

The *E. coli* BL21 was developed in the work of Studier and Moffatt (1986) after various modifications of the parental B cell line [136]. Today, it is the most widely used strain for recombinant expression. Along the way to BL21 development, several mutations were introduced. Some of them were beneficial for recombinant protein production, such as a mutation in the *hsdS* gene that prevents plasmid loss from transformed bacteria (introduced in the parental B834 strain; [137]). However, a few mutations may not have a direct correlation with recombinant production or may even hamper cultivation in autoinduction media, an alternative to IPTG induction. This is the case of the inactivation of *galK*, *galT* and *galE* genes, which encode important enzymes for galactose use and the Leloir pathway (introduced in the parental strain B707; [138] likewise, the absence of flagellar biosynthesis genes *fli*, which renders the non-motility in B lineage. However, in this instance, it also has the benefit of saving energy that might otherwise be spent on recombinant yield [131]. In addition, a major advantage of the B strain comes from the higher expression of genes related to amino acid synthesis and decreased expression of those for degradation, indicating their suitability for efficient protein production [139].

The BL21 cells carry knockouts in the *Lon* and *OmpT* genes, which encode cytoplasmic and outer membrane proteases, respectively. Those components consistently hinder recombinant manufacturing by hydrolyzing proteins throughout the downstream process. The BL21(DE3) is a derivative strain version that contains a λ prophage that encodes the T7 RNA polymerase, which recognizes the widely-used T7 promoter and is five to eight times faster compared to native *E. coli* polymerases [140, 141]. Consequently, BL21 is used solely for protein expression by *E. coli* native RNA polymerase promoters, e.g. lac, tac, trc, ParaBAD, PrhaBAD, and T5, upstream of the gene.

Several strains have been derived from the BL21 focusing on overcoming common challenges encountered in laboratory routine. Two key issues to bear in mind when creating RMPs, as discussed in the third section, are the codon usage ratio and protein folding errors. In addition to the bioinformatics tools mentioned below, there are specific *E. coli* strains developed to mitigate those potential issues. In this regard, an *E. coli* Rosetta-derived lineage was used to express RMPs designed for *T. cruzi, W. bancrofti,* and human HTLV detection [10, 115, 126]. This lineage harbors extra copies of genes encoding rare tRNAs, including for AUA, AGG, AGA, CUA, CCC, and GGA codons [142]. The strain BL21-CodonPlus(DE3)-RIL, which contains similar modifications, was also used to express the RMP rqTSA-25 for the diagnosis of bovine tapeworm [122]. These examples demonstrate the ability to address codon usage issues by selecting suitable lineages without entirely replacing the gene codons planning.

A problem with folding errors that is routinely described in the literature is the inclusion bodies occurrence due to misfolded recombinant proteins. Inclusion bodies are aggregates of biomolecules, mostly proteins, to which the bacteria become more susceptible during the recombinant expression [143]. The physicochemical properties of amino acids, particularly the hydrophobic interactions, are key factors that govern the formation of inclusion bodies [144]. As the RMPs have not undergone natural selection, they might exhibit instability issues, being more prone to form inclusion bodies. Moreover, protein expression at high rates also triggers inclusion body formation, which is a common feature of BL21 derivative strains. For instance, T7 promoters are able to raise recombinant proteins to constitute 50% of total cell proteins within a few hours [145]. The inclusion bodies form more readily under metabolic stress as the production of recombinant and host proteins compete for resources. This contest arises from the overload on DNA replication, the rivalry for transcription and translation elements, and the supplementary energy [146].

Three leading ways to avoid inclusion body formation without involving the RMP redesigning are (‘) reduce recombinant protein production, (2) use stress-adapted strains, or (3) insert certain adjustments into the RMP’s plasmids. The first approach mainly consists of culturing *E. coli* at lower temperatures (30° to 25 °C), slowing down expression, and enhancing protein stability [147, 148]. Furthermore, strains containing plasmids, such as pLysS or pLysE, significantly benefit RMP production by using T7 promoters, preventing inclusion body formation [149]. These plasmids co-express T7 lysozyme, which suppresses transcriptional leak of recombinant genes, an approach adopted for expression of the following RMPs: r-LMP, ITC 8.2, USM.TOXO1, rMEHB, and rMEHCV [2, 10, 19, 31, 40].

Another example that is in line with better-controlled transcription of recombinant genes is the adoption of *E. coli* Tuner(DE3) strain. This BL21-derivative carries a Lac permease enzyme mutation, ensuring uniform IPTG uptake across all cells and leading to concentration-dependent induction levels [150, 151]. Also, the Evo21(DE3) strain, a recently developed cell line adapted to recombinant expression burden, is a promising candidate for future RMP production. It expresses 3.6-fold higher levels than BL21(DE3) eight hours post-induction, also dealing better with inclusion body formation [152].

A key factor in the widespread use of *E. coli* is its remarkable ability to readily incorporate foreign DNA, especially in a plasmid format [153, 154]. Thus, several tags and vector arrangements have been developed to strategically improve recombinant yield, including through the mitigation of inclusion bodies. Regarding vectors, the pET series of expression plasmids is by far the most commonly used for recombinant research (> 220,000 published research studies cited its use; [155, 156]). Its first generation was developed using a pBR322 backbone. Over 100 derivatives have since been developed, with pET28a and pET15b being the most commonly used. These vectors enable fusion with the histidine-tag (His-tag), which is highly effective for detection in immunochemical assays, e.g., ELISA and Western blot, and for purification via immobilized metal-affinity chromatography [157]. The His-tag effectiveness is not distinct in either the N- or C-terminal junction. However, depending on the protein's folding, the tag may enter a cryptic pocket and lose its utility. Additionally, the C-terminal location can be useful when verifying protein integrity in electrophoretic assays, such as the Western blot.

However, the His-tag is unlikely to interfere with solubility, especially for large proteins and, therefore, does not prevent inclusion body formation. To promote protein solubility, researchers have used a range of fusion tags, including thioredoxin (Trx), glutathione S-transferase (GST), small ubiquitin-related modifier (SUMO), and maltose binding protein (MBP). Several vectors are available that carry these fusion tags [158]. The SUMO tag, which is added to the end of proteins that have their genes cloned in the pSUMO or pET SUMO vectors, is particularly effective in this regard. It can act as a chaperone and facilitate folding, as well as increasing solubility [159]. Moreover, there are vectors, such as pET43 and pET44/pET32, which carry N-utilization substance A (NusA) and Trx, respectively, as well as more specialized ones. such as pGEX and pMAL, which bring the tags GST and MBP, respectively.

The K-lineage *E. coli* strains are an alternative to the aforementioned B-lineage. Their isolation occurred in 1922 from the stool of a diphtheria patient in Palo Alto (CA, USA; [138, 160]), and since then, many strains adapted to recombinant expression have been developed. For the production of PALD, a RMP for human leprosy, the authors used the HMS-174 strain [41], and *E. coli* SG13009 was used for r-DME-G expression, a RMP for dengue detection [9]. Notably, these K-lineage bacteria outperform BL21 strains in lactose induction scenarios (autoinduction component). The HMS-174(DE3) strain exhibits a nearly threefold higher lactose uptake rate compared to BL21(DE3), and it accumulates less galactose, minimizing osmotic stress. As a result, the specific product titer was twice as high in the HMS-174 (DE3) strain, as compared to BL21(DE3) strain [161].

Considering all the advantages of using *E. coli* in the production of recombinant proteins, it is not surprising that the vast majority of studies with RMPs mentioned above used this host for the expression of their target antigens, highlighting the great biotechnological contribution of this microorganism to scientific research.

## Importance of bioinformatics for RMP analysis

Bioinformatics plays a crucial role in addressing challenges related to RMP production, particularly in cases of low heterologous overexpression and poor solubility [162, 163]. By employing bioinformatics tools, researchers can design optimized gene sequences for the target protein, including codon optimization to match the host organism's codon preferences [164, 165]. Codons are triplets of nucleotides in deoxyribonucleic acid (DNA) that code for specific amino acids in a protein [166]. Different organisms have variations in their codon usage preferences, with some being more frequently used than others [167]. When a gene from one organism is introduced into another, the differences in codon usage can lead to inefficient translation and lower protein expression levels [168, 169].

Bioinformatics tools analyze the codon usage patterns of both the source organism and the host organism [167, 170]. Researchers can then modify the gene sequence to replace rarely used codons with preferred codons of the host organism, a process known as codon optimization [171–173]. This adjustment improves the efficiency of translation and enhances protein expression levels in the host organism [171]. Besides, bioinformatics algorithms are used to identify the optimal codon replacements to maximize protein expression [174]. These algorithms consider factors such as codon frequency, transfer ribonucleic acid (tRNA) availability in the host organism, and messenger ribonucleic acid (mRNA) secondary structure [175]. By analyzing these factors, the bioinformatics tools generate a modified gene sequence that is more likely to be efficiently translated in the host [176]. Available bioinformatics tools for codon optimization and their features are shown in Table 6. Moreover, bioinformatics tools available from commercial entities can also provide gene synthesis services, being an additional option for researchers.

**Table 6:** Bioinformatics tools for codon optimization

Bioinformatics tool	Description
Gene designer	User-friendly software tool that allows researchers to design and optimize DNA sequences, including codon optimization. It provides a graphic interface for visualizing and editing gene sequences, and it can suggest codon substitutions based on codon usage preferences in the target organism [176]
JCat	Java Codon Adaptation Tool is an online tool that performs codon optimization for a target gene sequence. It takes into account the codon usage tables of both the source and target organisms and suggests codon replacements to enhance protein expression in the host organism [177]
Codon optimizer	Online tool that enables codon optimization for a wide range of organisms. Users can input their gene sequence and select the target organism, and the tool will provide a codon-optimized version of the gene [178]
DNA works	Software package that includes codon optimization functionality. It allows users to optimize gene sequences based on various criteria, such as codon frequency, secondary structure, and more [179]
COOL	Codon Optimization OnLine is an online tool that performs codon optimization for a variety of organisms. It provides options for specifying codon usage tables and other parameters to customize the optimization process [180]
OPTIMIZER	Program that optimizes codon usage based on a user-defined set of preferred codons. Researchers can input their codon usage table and specify codon preferences for optimization
ICOR	Codon optimization tool that uses recurrent neural networks to improve heterologous expression of synthetic genes [181]
EuGene	Comprehensive tool for analyzing and processing genetic information, ranging from codon usage to gene identification and structural analysis [182]
COStar	Algorithm that uses D-star Lite to address codon optimization by creating a specialized graph structure [183]
D-Tailor	DNA-Tailor is a versatile tool for designing DNA sequences with specific characteristics, using a customizable Monte Carlo approach [184]
CODA	Computationally Optimized DNA Assembly is an algorithm that leverages the redundancy in the genetic code to generate overlapping oligonucleotides with specific thermodynamic properties [185]
ATGme	Open-source web-based application that simplifies optimization through its user-friendly interface, offering three strategies: one-click, bulk based on codon types, and individualized custom optimization [185]
Codon wizard	Versatile tool for analyzing and optimizing codon usage in various types of input sequences. It allows users to freely combine algorithms, including both established and novel ones, to achieve desired results [186]

Additionally, bioinformatics can predict and identify regions of the protein prone to misfolding or aggregation, enabling the design of modifications or fusion tags that enhance protein solubility and stability [187, 188]. Bioinformatics tools and algorithms analyze the amino acid sequence of a protein to predict regions that are susceptible to misfolding or aggregation [189]. These regions are often characterized by sequences that have a high propensity to form beta-sheet structures or expose hydrophobic residues [190]. Bioinformatics methods use algorithms and databases that incorporate data on protein structures, folding kinetics, and known aggregation-prone motifs [191, 192]. Once problematic regions are identified, bioinformatics can aid in the design of modifications to mitigate misfolding and aggregation issues [193]. One common approach is to introduce point mutations that disrupt or stabilize specific interactions within the protein structure [193, 194]. For example, mutations can be introduced to reduce the exposure of hydrophobic residues or promote more favorable intramolecular interactions [195].

Bioinformatics tools can also predict the impact of these mutations on the protein's stability and solubility. They guide the selection of fusion tags or chaperone proteins that enhance protein solubility and stability [188, 196]. Fusion tags are peptide sequences added to the target protein that improve its expression and solubility [197]. Bioinformatics helps choose appropriate fusion tags by considering such factors as size, charge, and affinity for purification [198]. Conversely, chaperone proteins can assist in the correct folding of the target protein [199]. Computational tools can suggest chaperone proteins known to interact with proteins of interest, facilitating proper folding during expression [200, 201]. Advanced bioinformatics tools can also perform in silico structural modeling to simulate how modifications or fusion tags will affect the protein's three-dimensional structure [202]. Available bioinformatic tools to predict misfolding or aggregation and their features are shown in Table 7.

**Table 7:** Bioinformatics tools to predict misfolding or aggregation and design modifications or fusion

Bioinformatics tool	Description
TANGO	Bioinformatics tool that predicts amyloidogenic regions in protein sequences. It calculates the aggregation propensity of different segments of a protein and helps identify regions that are likely to form aggregates. Researchers can use this information to design modifications to mitigate aggregation risks [203]
AGGRESCAN	Bioinformatics tool that predicts aggregation-prone regions in protein sequences based on the physicochemical properties of amino acids. It calculates aggregation propensity scores and identifies potential hotspots for aggregation [204]
FoldX	While primarily a tool for protein stability prediction, FoldX can be used to assess the impact of mutations on protein folding and aggregation propensity. Researchers can use it to evaluate the effects of mutations designed to enhance solubility and stability [205]
Rosetta FlexPepDock	Versatile suite of software tools for protein structure prediction and design. It can be used to model protein structures and assess the impact of mutations or modifications on protein stability and solubility [206]
I-TASSER	Protein structure and function prediction tool that can identify potential aggregation-prone regions based on structural modeling. It provides insights into the three-dimensional structure of a protein and can help design modifications to improve stability [207]
TIsigner	Computational tool in bioinformatics that is used to design and optimize translation start sites (TIS) in gene or mRNA sequences, allowing control and adjustment of gene expression [208]
SoDoPE	Valuable tool in protein engineering and recombinant protein production. It allows users to analyze a protein sequence and its domains to predict and enhance solubility [208]
ESPRESSO	System or tool designed to estimate the expression and solubility of proteins in different expression systems. Uses data analysis and bioinformatics approaches to predict the ability of a protein to be expressed and maintained in a soluble form in expression systems such as bacteria, yeast, or mammalian cells [209]
Aggrescan3D	Web server used to predict the aggregation properties of protein structures. It achieves this by incorporating 3D structural information and assessing the significance of solvent-exposed aggregation-prone regions [210]

Moreover, bioinformatics analyses aid in selecting the most suitable host organism and expression system based on the protein's characteristics, ultimately streamlining the production process [155, 211]. Bioinformatics helps researchers choose the optimal host organism by considering factors such as the protein's size, complexity, post-translational modifications, the organism's genetic tools and available resources, and the desired protein yield [176, 212, 213]. For example, if a protein requires complex glycosylation, bioinformatics analysis may suggest using a eukaryotic expression system such as yeast or mammalian cells [214]. Conversely, if a simpler prokaryotic system, like *E. coli*, is sufficient, bioinformatics could confirm this choice based on the protein's features [162, 215].

Bioinformatics can assist in selecting the most suitable promoter for gene expression in the chosen host organism. By analyzing promoter databases and regulatory elements, researchers can identify promoters that match the protein's expression requirements, including inducible or constitutive expression, high-level production, or tissue-specific expression [216, 217]. In the case of secreted proteins, computational tools can predict signal peptides that target the protein for secretion in host organisms, such as yeast or bacteria. This ensures efficient secretion into the extracellular space [218, 219]. Bioinformatics tools can aid in designing plasmids for cloning and expression. This includes selecting appropriate vectors, resistance markers, and other genetic elements necessary for gene expression. Optimized plasmid design can enhance protein production efficiency [156, 220]. In addition, possible post-translational modifications, such as glycosylation or phosphorylation sites, can be predicted, allowing researchers to plan appropriate quality control and subsequent processing steps [221–223]. Available bioinformatic tools to select the most suitable host organism for RMPs expression and their features are shown in Table 8.

**Table 8:** Bioinformatic tools to select the most suitable host organism and expression system

Bioinformatics tool	Description
Host designer	Web-based tool that assists in choosing the most appropriate host organism for recombinant protein expression based on user-defined criteria and protein characteristics [224]
Vector NTI	This software offers features for sequence analysis and vector design, aiding in the selection of host organisms and plasmids for protein expression [225]
Geno CAD	Web-based platform that assists in designing genetic constructs, including promoter selection, for recombinant protein expression in various hosts [226]
Regulon DB	Database containing information on transcriptional regulation in *Escherichia coli*, aiding in promoter selection for bacterial expression [227]
Signal P	Tool for predicting signal peptides in protein sequences, which is essential for secreted protein expression [228]
Deep Sig	Tool for predicting signal peptides and their cleavage sites in proteins [229]
ApE	Versatile multi-platform application used to design plasmids and other constructs by simulating cloning methods such as PCR, Gibson assembly, restriction-ligation assembly, and Golden Gate assembly in silico [230]
Plas mapper	Web server that enables users to create, modify, add annotations, and interactively display high-quality plasmid maps [231]
Opt flux	Computational tool for metabolic engineering and optimization that can be used to predict optimal conditions for recombinant protein production [232]
Phospho site	Web-based bioinformatics resource specifically dedicated to cataloging the sites where proteins undergo phosphorylation in humans and mice [233]

In summary, by leveraging the power of computational techniques and data-driven insights, the field of bioinformatics plays a crucial role in the synthesis of RMPs. It acts as a vital link between genetic engineering and protein manufacturing, providing detailed answers to some of the most difficult problems involved in this procedure. Bioinformatics enables researchers to make informed choices about host organisms, expression systems, and modifications, ensuring that proteins are effectively synthesized, correctly folded, and easily soluble. Codon optimization, structural analysis, and predictive modeling provide these choices by speeding up the production process and raising the likelihood of successfully producing functional recombinant proteins overall. The biotechnology and pharmaceutical sectors continue to rely on the adaptability of recombinant proteins for therapeutic and commercial applications, and bioinformatics continues to play a crucial role in advancing innovation and the ability to fully realize the potential of these proteins for the advancement of science and society.

## Importance of biophysical analysis of the RMP´s structure for diagnosis

Biophysical analysis, in general, is applied to characterize biological systems, providing important information on the conformational stability of the molecules. These techniques have several advantages, such as obtaining structural information about the protein of interest in the most diverse experimental conditions. Normally, these techniques require a small amount of sample and provide rapid data acquisition [234, 235]. Among these techniques, circular dichroism (CD) can be applied for the characterization of chiral systems, including peptides, proteins, carbohydrates, and nucleic acids, based on the optical phenomenon of absorption of circularly polarized light. The study of proteins using this technique allows the identification and estimation of secondary structures and provides information about the tertiary structure from the Far-UltraViolet (Far-UV) and near-Ultra-Violet (near-UV) CD spectra, respectively. Furthermore, conformational changes can be analyzed under different experimental conditions, such as pH, temperature, and salt concentration in the solvent, as well as interactions between protein-protein, protein-membrane, or several other molecules [235–239].

The CD technique has been applied in several studies to estimate the secondary structure profile of the RMPs in solution for application in diagnostic tests [2, 4, 19, 107, 114]. Typically, the RMPs are rationally designed containing solvent-exposed bound epitopes. Therefore, to determine the secondary structure content of the alpha helix, beta-sheet, beta-turn, and random coil, the Far-UV spectra of the RMPs are recorded in solution, and the ellipticities are converted to molar ellipticity [θ] (deg.cm^2^/dmol) [240, 241]. These results can be used to confirm whether the RMPs recombinant preserved the predicted secondary structure or defined the stability under different environments.

Several RMPs developed for disease diagnosis, such as rMEHB for hepatitis B [19], rMEHCMV for human cytomegalovirus [107], rMERUB for rubella [114], and rMELEISH for visceral canine and human leishmaniasis [4], showed a Far-UV CD spectra with a pronounced negative dichroic band at 200 nm, compatible with an unordered structure (≥ 40%), followed by β-sheets content (≥ 35%) and a lower α-helix percentage (≤ 15%). In contrast, rMEHCV developed for hepatitis C diagnosis showed a negative dichroic band at 208 nm, indicating a higher content of β-sheets (≥ 56%) [2]. In these studies, CD results showed that proteins presented structural stability at 25 °C under neutral and basic pH. In addition, changes in the CD signal as a function of temperature ranging from 25 to 95 °C indicated that the structural stability of proteins decreases at temperature above 40 °C [2, 19, 107]. Taken together, the results from the biophysical analysis were fundamental to establishing the ideal structural conditions of the RMP, in which their biological functions would be preserved.

It is known that greater exposure of epitopes would be the ideal condition for better recognition of the molecule by antibodies present in serum [1]. Therefore, the structural study of RMPs in solution is one of the most important steps for evaluating their diagnostic capacity, being a useful tool aimed at increasing the efficiency of diagnostic tests, since the CD technique is an effective strategy for determining the secondary structure and folding properties of proteins (Greenfield, 2009).

## Conclusion

In the present review, since only studies using the nomenclature “recombinant multiepitope protein” were selected, despite of the large number of studies using RMPs for immunodiagnostics, this number may be underestimated, since some authors use the nomenclature “chimera” instead of RMP. Ever since the first description of the development and use of RMPs for immunodiagnostics, these molecules have been widely used in the diagnosis of multiple animal and human diseases. It is evident that the use of RMPs for immunological diagnosis has increased significantly over the past ten years, most likely as a result of the benefits they have over other diagnostic kits that have been covered here. In reality, they are molecules of considerable scientific interest due to the high sensitivity and specificity values they offer in immunological diagnoses. In this regard, a significant amount of the next generation of diagnostic technologies will likely include these compounds.

## Data Availability

Not applicable.
